# La dégénérescence cérébelleuse paranéoplasique révélant une récidive métastatique du cancer d’ovaire

**DOI:** 10.11604/pamj.2019.33.281.17711

**Published:** 2019-08-02

**Authors:** Othmane Zouiten, Zineb Benbrahim, Lamiae Amaadour, Nawfel Mellas

**Affiliations:** 1 Service d’Oncologie Médicale, CHU Hassan II, Route de Sidi Harazem, Fès, Morocco

**Keywords:** La dégénérescence cérébelleuse subaigue, anticorps onconeuronaux, syndrome neurologique paranéoplasique, Subacute cerebellar degeneration, onconeural antibodies, paraneoplastic neurologic degeneration

## Abstract

Le syndrome neurologique paranéoplasique (SNP) est caractérisé par l'apparition aigue et subaigue d'un syndrome neurologique associé à un cancer actif ou infraclinique. C'est une manifestation rare dont le diagnostic précoce modifie le pronostic neurologique et carcinologique. Parmi les SNP on distingue la dégénérescence cérébelleuse subaiguë souvent associée à un cancer gynécologique ou mammaire. Nous rapportons le cas d'une patiente de 50 ans suivie pour un adénocarcinome d'ovaire opéré et dont la récidive s'est révélée par une dégénérescence cérébelleuse subaiguë avec des AC anti Yo positifs.

## Abstract (en)

Paraneoplastic neurologic degeneration (PND) is characterized by acute and subacute onset neurological syndrome associated with active or with subclinical cancer. It is rare but early diagnosis can improve the neurological and carcinological prognosis. Among the PNDs it is possible to distinguish subacute cerebellar degeneration often associated with gynecological or breast cancer. We report the case of a 50-year old female patient on follow-up for ovarian adenocarcinoma treated with surgery and whose recurrence was detected due to anti-Yo antibody-positive subacute cerebellar degeneration.

## Introduction

Le syndrome neurologique paranéoplasique (SNP) est défini par la survenue d'un syndrome neurologique qui ne peut être expliqué par une étiologie métastatique, iatrogène, toxique ou carentielle. On distingue plusieurs présentations cliniques dont les plus rapportées en littérature sont: la dégénérescence cérébelleuse paranéoplasique (DCP), l'encéphalite limbique, l'encéphalomyélite, la neuropathie sensitive subaiguë, le syndrome de Lambert Eaton, l'opsoclonus-myoclonus, la dermato-polymyosite et le syndrome pseudo-occlusif. La (DCP) complique de nombreuses tumeurs comme le cancer du sein, du poumon et certains cancers gynécologiques. Nous rapportons le cas d'une patiente ayant présenté un syndrome cérébelleux avec des anticorps anti-Yo positifs révélant une récidive infra radiologique d'un cancer de l'ovaire.

## Patient et observation

**Figure 1:**
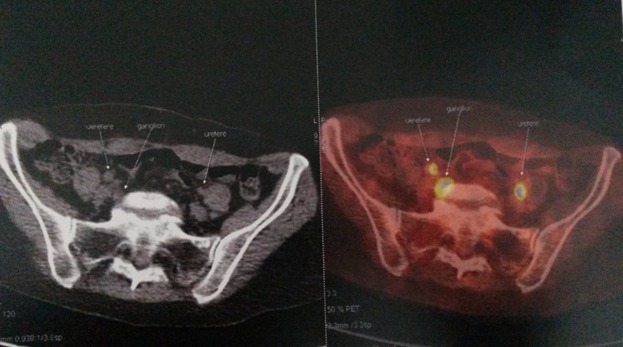
Ganglions présacrés hypermétaboliques documentant la récidive du cancer d’ovaire

Il s'agit d'une patiente de 50 ans suivie pour asthme évoluant depuis l'âge de 20 ans et qui se présente en Aout 2015 pour des douleurs abdomino-pelviennes évoluant dans un contexte d'amaigrissement. L'examen abdominal trouve une ascite de moyenne abondance sans autres anomalies cliniques décelables. Une tomodensitométrie thoraco-abdominopelvienne a montré deux masses latéro-utérines solido-kystiques faisant évoquer une tumeur ovarienne bilatérale associée à une carcinose péritonéale. Le CA125 était à 25 504 UI/ml. Une biopsie péritonéale a été réalisée. L'étude anatomopathologique revient en faveur d'une métastase péritonéale, d'un adénocarcinome moyennement différencié compatible avec une origine ovarienne. Un traitement chirurgical optimal n'a pas pu être assuré d'emblée vu l'étendue de la maladie. Une chimiothérapie première était administrée associant carboplatine et paclitaxel. Après 8 cures, la patiente a subit une hystérectomie totale, une annexectomie bilatérale, une omentectomie, un curage pelvien lomboaortique et des biopsies multiples des gouttières pariétocoliques. L'étude anatomopathologique de la pièce révèle un adénocarcinome séreux ovarien bilatéral stade Ib. Les suites post opératoires étaient simples. Un suivi régulier a été fait. Après 8 mois, la patiente consulte pour une instabilité à la marche avec des crises de vertiges rotatoires d'aggravation progressive. L'examen trouve une patiente avec un index de karnofsky <60%, à l'examen neurologique une dysarthrie, une marche ataxique, élargissement du polygone de sustentation, avec une manœuvre doigt nez positive, à l'examen abdominal pas de masse ni de distension abdominale. Le dosage de CA125 a augmenté à 357 UI/ml par rapport au nadir à 122 UI/ml en post opératoire. La tomodensitométrie cérébro-thoraco-abdominopelvienne revient normale ainsi que l'IRM cérébrale, la ponction lombaire et le bilan infectieux et carentiel. Devant la suspicion d'une atteinte auto-immune, on a procédé à la recherche des anticorps onconeuronaux dans le sang. Le taux des anticorps anti-Yo était élevé. Ainsi la dégénérescence cérébelleuse subaiguë paranéoplasique était retenue. Un pet scanner a été réalisé à la recherche de la récidive tumorale et qui a montré une nodule inter spléno-gastrique hypermétabolique (SUV max = 7,1) mesurant 25*15 mm avec des ganglions hypermétaboliques (SUV max = 3.23) présacrés latéralisés à gauche dont le plus grand mesure 8 mm très suspect de récidive métastatique (Figure 1). La chimiothérapie par carboplatine et paclitaxel a été reprise. Après un total de 6 cures la patiente a présenté une stabilité clinique et biologique avec baisse des anticorps anti-Yo.

## Discussion

Les syndromes neurologiques paranéoplasiques (SNP) sont des syndromes neurologiques rares associés à un cancer et par définition ne s'expliquant pas par une cause métastatique, carentielle, métabolique, infectieuse ou iatrogène [1]. Cette affection est responsable de plusieurs formes cliniques variables selon la localisation de la lésion qui peut intéresser soit le système nerveux centrale, soit le système nerveux périphérique, ou la jonction neuro-musculaire [2]. La DCP est associée le plus souvent à des cancers pulmonaires et gynécologiques dont l'incidence et la prévalence restent inconnues. Il touche aussi bien les femmes que les hommes. L'âge moyen de survenue des DCP reflète l'âge de survenue des cancers [3]. Le délai d'apparition des manifestations cliniques par rapport à la tumeur primitive est variable pouvant aller de quelques mois en quelques années comme elles peuvent être un mode révélateur de la tumeur. La symptomatologie est associée à un syndrome cérébelleux statique et cinétique bilatéral et une dysarthrie. Un syndrome vertigineux et un nystagmus peuvent être observés [4]. L'imagerie radiologique cérébrale est généralement normale au début comme dans notre cas. D'autres signes radiologiques peuvent apparaître après plusieurs mois à des années comme l'atrophie cérébelleuse avec une dilatation du quatrième ventricule, sans atteinte du tronc cérébral [5, 6]. La physiopathologie de la DCP est mal étudiée. L'hypothèse d'un mécanisme auto-immun en cause a été suggérée. On estime qu'il s'agit d'une réaction croisée due à l'expression ectopique par la tumeur de protéines normalement exprimées par le système nerveux. La présence d'auto-anticorps circulants (sérum et liquide céphalorachidien (LCR)), spécifiquement associés aux SNP, est une des caractéristiques de ces syndromes. On distingue 2 types de SNP selon la cible des anticorps qui leur sont associés et qui peuvent être dirigés contre des cibles intracellulaires (onconeuronaux) ou membranaires. Parmi les anticorps onconeuronaux identifiés dans la DCP on trouve: Hu, Yo, CV2, Ri, Ma, Tr/DNER, amphiphysine, Sox1 [6]. Les anticorps anti-Yo sont associés à certains cancers gynécologiques (ovaire et rarement utérus), mammaire, pulmonaire et gastrique. La présence d'anticorps anti-Yo chez une femme présentant un syndrome cérébelleux est dans 90% des cas associée à un cancer du sein ou de l'ovaire. Ces anticorps reconnaissent une protéine neuronale exprimée dans le cytoplasme des cellules de Purkinje du cervelet appelée CDR2 exprimée également dans les tumeurs du sein et des ovaires. La découverte de ces anticorps doit inciter à rechercher ce type de tumeurs [7]. Le traitement des DCP repose sur la prise en charge de la tumeur primitive. Les immunosuppresseurs ou immunomodulateurs n'ont pas montré d'efficacité dans cette indication [8]. Le pronostic de la DCP est souvent péjoratif et il est lié à la persistance des signes cliniques malgré un traitement anti-tumoral adapté. La survie est d'environ 22 mois pour les cancers gynécologiques. Sur le plan fonctionnel 10% des patients peuvent marcher sans assistance [8, 9].

## Conclusion

Chez une patiente présentant un syndrome cérébelleux associé à des AC anti Yo positifs, la recherche active d'un cancer gynécologique doit être réalisée. Le recours au Pet scanner est souvent nécessaire afin de dépister des cancers au stade infraclinique et infraradiologique.

## Conflits d’intérêts

Les auteurs ne déclarent aucun conflit d'intérêts.
